# Personalized anticoagulant therapy in venous thromboembolism: current role of direct oral anticoagulants, unmet clinical needs, and future perspectives on Factor XI inhibition

**DOI:** 10.3389/fphar.2026.1959476

**Published:** 2026-09-30

**Authors:** Carmine Siniscalchi, Alessandro Perrella, Ugo Trama, Manuela Basaglia, Francesca Futura Bernardi, Pierpaolo Di Micco

**Affiliations:** 1 Internal Medicine Department, Parma University Hospital, Parma, Italy; 2 Emerging Infectious Disease and High Contagiousness Unit P.O. D. Cotugno-AORN Ospedali dei Colli, Naples, Italy; 3 Regional Observatory for Infectious Disease, Naples, Italy; 4 Coordination of the Regional Health System, General Directorate for Health Protection, Naples, Italy; 5 Internal Medicine Ward “P.O. Santa Maria delle Grazie”, Naples, Italy

**Keywords:** anticoagulant therapy, biomarkers, bleeding risk, direct oral anticoagulants, Factor XI inhibition, real-world evidence, statins, venous thromboembolism

## Abstract

Venous thromboembolism (VTE), encompassing deep vein thrombosis and pulmonary embolism, remains a major cause of morbidity and mortality worldwide. The introduction of direct oral anticoagulants (DOACs) has transformed the management of VTE by providing effective and convenient alternatives to vitamin K antagonists. However, despite their favorable efficacy–safety profile, clinically relevant bleeding, treatment individualization, and the management of patients with competing thrombotic and hemorrhagic risks remain important unmet clinical needs. Contemporary VTE management increasingly requires a personalized approach integrating clinical characteristics, comorbidities, biomarkers, and real-world evidence to optimize anticoagulant selection, treatment intensity, and duration. Large prospective registries have expanded our understanding of VTE across heterogeneous clinical settings, including cancer-associated thrombosis, postoperative VTE, incidental and subsegmental pulmonary embolism, and other high-risk populations frequently underrepresented in randomized clinical trials. In parallel, emerging prognostic tools, including D-dimer, hemoglobin, inflammatory indices, lipid-related biomarkers, and machine-learning-based prediction models, are contributing to a more refined assessment of thrombotic and bleeding risk. These emerging therapies are being evaluated as potential strategies to reduce bleeding while maintaining antithrombotic efficacy; however, recent phase III findings have demonstrated heterogeneous efficacy across clinical indications, emphasizing that their future role is likely to be indication-specific. This narrative review examines the current role of DOACs in VTE management, discusses the clinical and biological determinants of individualized anticoagulant therapy, and critically appraises the evolving and heterogeneous evidence for Factor XI inhibition as a potential future anticoagulant strategy.

## 1 Introduction

Venous thromboembolism (VTE), including deep vein thrombosis (DVT) and pulmonary embolism (PE), remains one of the leading causes of cardiovascular morbidity and mortality worldwide despite substantial advances in diagnostic strategies and anticoagulant therapies (Goldhaber and Bounameaux, 2012; Heit, 2015; Di Nisio et al., 2016; Silverstein et al., 1998). The clinical presentation and prognosis of VTE are highly heterogeneous, ranging from asymptomatic distal thrombosis to life-threatening PE associated with hemodynamic instability and multiorgan dysfunction (Goldhaber and Bounameaux, 2012; Silverstein et al., 1998). Anticoagulant therapy remains the cornerstone of VTE treatment across this broad clinical spectrum. However, treatment intensity, pharmacological choice, and duration must be balanced against the individual risk of recurrent thrombosis and bleeding.

Over the last decade, increasing attention has been directed toward individualized approaches to VTE management integrating clinical characteristics, laboratory biomarkers, imaging findings, and patient-related risk factors (Kearon et al., 2016; Ortel et al., 2020; Konstantinides et al., 2019; Stevens et al., 2021). The introduction of direct oral anticoagulants (DOACs) has substantially changed VTE management by providing fixed-dose regimens, rapid onset of action, predictable pharmacokinetics, and fewer food and drug interactions than vitamin K antagonists. Randomized trials have established their efficacy and generally favorable safety profile in appropriately selected patients. Nevertheless, DOAC therapy does not eliminate the risk of major or clinically relevant non-major bleeding, and important uncertainties remain in older and frail patients, individuals with severe renal dysfunction, active cancer, extreme body weight, clinically relevant drug interactions, or competing thrombotic and hemorrhagic risks. Current international guidelines recommend risk-adapted strategies for anticoagulant selection, treatment duration, and follow-up according to recurrence risk, bleeding risk, cancer status, renal dysfunction, and comorbidity burden (Kearon et al., 2016; Ortel et al., 2020).

Large prospective registries have complemented randomized clinical trials by providing evidence on anticoagulant effectiveness, safety, and treatment patterns in populations frequently underrepresented in pivotal studies. Among these, the Registro Informatizado de la Enfermedad TromboEmbólica (RIETE) has contributed important real-world data on patients with cancer-associated thrombosis, incidental and subsegmental pulmonary embolism, postoperative VTE, advanced age, multiple comorbidities, and competing bleeding risks (Mastroiacovo et al., 2019; Fernández-Capitán et al., 2020; Peris et al., 2021; Bikdeli et al., 2021; Dubois-Silva et al., 2023; Siddiqui et al., 2024; Avnery et al., 2020; Mora et al., 2023; Golemi et al., 2020; Riera-Mestre et al., 2019; Expósito-Ruiz et al., 2021; Bikdeli et al., 2022; Mellado et al., 2019). These observations are particularly relevant when evaluating the generalizability of DOAC trials and identifying clinical phenotypes in which standard anticoagulant strategies may provide an uncertain net clinical benefit.

Nevertheless, registry-based evidence should be interpreted cautiously because of residual confounding, selection bias, and heterogeneity in clinical management. In parallel, biomarkers, clinical prediction rules, and machine-learning approaches have been investigated to improve the assessment of recurrent VTE and bleeding during anticoagulant treatment (Siddiqui et al., 2024; Avnery et al., 2020; Mora et al., 2023; Golemi et al., 2020). Although prospective validation remains limited for several emerging tools, their integration with clinical characteristics may help refine decisions regarding anticoagulant selection, dose, duration, and follow-up intensity.

Despite the favorable benefit–risk profile of DOACs, bleeding remains the principal limitation of long-term anticoagulant therapy. This unmet need has stimulated the development of agents targeting coagulation factors that may contribute predominantly to thrombus propagation while having a more limited role in physiological hemostasis. Factor XI and activated Factor XI have emerged as particularly attractive targets because congenital Factor XI deficiency is generally associated with a milder and less predictable bleeding phenotype than deficiencies involving components of the common coagulation pathway. Pharmacological inhibition of Factor XI or Factor XIa is therefore being investigated as a potential strategy to preserve antithrombotic efficacy while reducing the risk of bleeding. However, the clinical role of these agents, their comparative efficacy against established DOACs, and the patient populations most likely to benefit remain to be defined.

Alongside advances in anticoagulant pharmacology, non-anticoagulant adjunctive strategies have also attracted interest. Statins have been investigated because of their anti-inflammatory, endothelial-stabilizing, and potential antithrombotic effects (Siniscalchi et al., 2023). Observational studies have suggested associations between statin use and improved outcomes in selected VTE populations (Siniscalchi et al., 2024; Siniscalchi et al., 2022). However, randomized evidence remains limited, causality cannot be established, and statins should be regarded as potential complementary interventions rather than alternatives to established anticoagulant therapy.

Several reviews and international guidelines have addressed the efficacy of individual anticoagulant classes, the management of specific VTE populations, or the prediction of recurrent and bleeding events (Kearon et al., 2016; Ortel et al., 2020; Konstantinides et al., 2019; Stevens et al., 2021). However, fewer reviews have integrated the established role of DOACs, their residual clinical limitations, real-world evidence, patient-specific biological vulnerability, and the emerging development of Factor XI-directed therapies within a single personalized treatment framework.

The aim of this narrative review is to examine the evolution of personalized anticoagulant therapy in VTE, with particular emphasis on the current role and residual limitations of DOACs. We discuss how clinical phenotypes, comorbidities, biomarkers, predictive models, and real-world evidence may support individualized decisions regarding anticoagulant choice, intensity, and duration. We also review the biological rationale and emerging clinical evidence for Factor XI and Factor XIa inhibition as potential future anticoagulant strategies, while considering the complementary and still investigational role of non-anticoagulant adjunctive therapies.

### 1.1 Literature search strategy and study selection

This narrative review was conducted using a structured, though non-systematic, approach to identify relevant literature on personalized management of venous thromboembolism (VTE). Accordingly, this review should be interpreted as a structured narrative synthesis of the literature rather than a formal systematic review. A comprehensive search was performed in PubMed/MEDLINE and Embase databases from database inception to August 2026. Search terms included combinations of ‘venous thromboembolism’, ‘pulmonary embolism’, ‘deep vein thrombosis’, ‘direct oral anticoagulants’, ‘DOAC’, ‘Factor XI’, ‘Factor XIa’, ‘FXI’, ‘FXIa’, ‘Factor XI activity’, ‘Factor XI inhibitor’, ‘Factor XIa inhibitor’, ‘abelacimab’, ‘milvexian’, ‘asundexian’, ‘personalized medicine’, ‘risk stratification’, ‘biomarkers’, ‘D-dimer’, ‘thrombophilia’, ‘Factor V Leiden’, ‘prothrombin G20210A’, ‘thrombin generation’, ‘global haemostasis’, ‘global hemostasis’, ‘bleeding risk’, ‘bleeding prediction’, ‘VTE-BLEED’, ‘machine learning’, ‘LDL cholesterol’, and ‘statins’. Reference lists of relevant reviews, randomized clinical trials, and guidelines were also screened to identify additional pertinent studies.

## 2 Direct oral anticoagulants in contemporary VTE management

### 2.1 Pharmacological background

Direct oral anticoagulants (DOACs) have profoundly changed the treatment of venous thromboembolism (VTE), progressively replacing vitamin K antagonists (VKAs) as the preferred anticoagulant strategy in most eligible patients. Unlike VKAs, which indirectly reduce the synthesis of multiple vitamin K-dependent coagulation factors, DOACs selectively inhibit a single activated coagulation factor. Dabigatran is a direct thrombin inhibitor, whereas rivaroxaban, apixaban, and edoxaban directly inhibit activated Factor X (FXa). These agents are characterized by a rapid onset of action, relatively predictable pharmacokinetic and pharmacodynamic profiles, fixed-dose administration, and fewer food and drug interactions than VKAs, thereby eliminating the need for routine coagulation monitoring in most patients (van Es et al., 2014).

Despite these shared characteristics, clinically relevant pharmacological differences exist among individual DOACs. These include dosing frequency, renal elimination, metabolism through cytochrome P450 pathways, interaction with P-glycoprotein, and the requirement for initial parenteral anticoagulation. Rivaroxaban and apixaban can be administered as single-drug strategies from the beginning of acute VTE treatment, using an initial intensified oral regimen. Conversely, dabigatran and edoxaban require an initial course of parenteral anticoagulation before oral treatment is started (Schulman et al., 2009; EINSTEIN Investigators et al., 2010; Investigators et al., 2012; Agnelli et al., 2013a; Investigators et al., 2013). Accordingly, the selection of a specific DOAC should take into account renal and hepatic function, age, body weight, concomitant medications, cancer status, gastrointestinal bleeding risk, anticipated adherence, and the expected duration of anticoagulant therapy.

The introduction of DOACs has simplified VTE treatment while preserving antithrombotic efficacy. However, the pharmacological inhibition of thrombin or FXa inevitably affects physiological hemostasis as well as pathological thrombosis. Consequently, no currently available DOAC completely separates protection against thromboembolism from the risk of bleeding.

### 2.2 Clinical evidence supporting DOACs in VTE

The efficacy and safety of DOACs for the treatment of acute VTE have been evaluated in large phase III randomized clinical trials. In the RE-COVER study, dabigatran, administered after initial parenteral anticoagulation, was non-inferior to warfarin for the prevention of recurrent symptomatic VTE, with a similar rate of major bleeding and a lower rate of major or clinically relevant non-major bleeding (Schulman et al., 2009). Rivaroxaban was evaluated as a single-drug strategy in the EINSTEIN programme. It showed efficacy comparable to conventional treatment in patients with acute deep vein thrombosis and acute pulmonary embolism, with a simplified regimen that avoided prolonged initial treatment with parenteral anticoagulants (EINSTEIN Investigators et al., 2010; Investigators et al., 2012).

Similarly, the AMPLIFY trial demonstrated that a fixed-dose, single-drug regimen of apixaban was non-inferior to conventional therapy with enoxaparin followed by warfarin and was associated with significantly less major bleeding (Agnelli et al., 2013a). In the Hokusai-VTE trial, edoxaban, administered after initial heparin treatment, was non-inferior to warfarin for the prevention of recurrent symptomatic VTE and was associated with a lower incidence of the composite of major and clinically relevant non-major bleeding (Investigators et al., 2013). Taken together, the pivotal phase III trials established that DOACs provide efficacy comparable to VKAs, with a generally favorable safety profile and greater practical convenience (van Es et al., 2014; Schulman et al., 2009; EINSTEIN Investigators et al., 2010; Investigators et al., 2012; Agnelli et al., 2013a; Investigators et al., 2013).

DOACs have also expanded therapeutic options for patients requiring anticoagulation beyond the initial treatment period. In the AMPLIFY-EXT trial, both the standard and reduced doses of apixaban substantially reduced recurrent VTE compared with placebo without a significant increase in major bleeding (Agnelli et al., 2013b). In the EINSTEIN CHOICE trial, rivaroxaban administered at either 20 mg or 10 mg once daily was more effective than aspirin in preventing recurrent VTE, with similarly low rates of major bleeding (Weitz et al., 2017). These findings support the use of reduced-dose regimens of selected DOACs during the extended-treatment phase in appropriately selected patients.

The availability of full-dose and reduced-dose regimens has facilitated a more individualized approach to long-term anticoagulation. Nevertheless, decisions regarding treatment extension should not be based exclusively on drug availability or convenience. They require a careful assessment of the expected risk of recurrent thrombosis, the individual risk of bleeding, patient preferences, comorbidities, and the clinical consequences of both recurrent VTE and hemorrhagic complications.

### 2.3 Residual limitations and unmet needs of DOAC therapy

Despite representing a major therapeutic advance, DOACs have not eliminated the principal limitations of anticoagulant treatment. Bleeding remains the most important complication of both initial and extended anticoagulation and frequently determines whether treatment is continued, reduced, interrupted, or permanently discontinued. Although pivotal trials generally demonstrated a favorable safety profile compared with VKAs, major bleeding and clinically relevant non-major bleeding continue to occur, particularly in patients with increased biological or clinical vulnerability (van Es et al., 2014; Schulman et al., 2009; EINSTEIN Investigators et al., 2010; Investigators et al., 2012; Agnelli et al., 2013a; Investigators et al., 2013; Agnelli et al., 2013b; Weitz et al., 2017).

Several patient populations were underrepresented or excluded from the pivotal DOAC trials. These include patients with severe renal dysfunction, advanced frailty, very low or very high body weight, clinically relevant thrombocytopenia, severe liver disease, pregnancy, and complex multimorbidity. Additional uncertainty concerns patients with antiphospholipid syndrome, individuals receiving strong P-glycoprotein or cytochrome P450 modulators, and patients with active malignancies associated with a high risk of gastrointestinal or genitourinary bleeding.

Renal function represents a particularly important determinant of DOAC exposure because all available agents undergo renal elimination to varying degrees. Progressive or acute deterioration of renal function may therefore increase anticoagulant exposure and bleeding risk. Conversely, inappropriate dose reduction motivated by concerns regarding age, frailty, or bleeding may reduce antithrombotic protection. These competing risks highlight the limitations of standardized dosing approaches in clinically heterogeneous populations.

The relatively short half-lives of DOACs offer advantages when treatment must be interrupted, but they also make adherence essential. Missing even a limited number of doses may result in a rapid reduction in anticoagulant protection. Furthermore, clinically relevant drug interactions, the management of urgent invasive procedures, the availability and cost of reversal agents, and uncertainty regarding optimal treatment after recurrent VTE during apparently adequate therapy remain important practical challenges.

The residual risk of bleeding is especially relevant in patients requiring indefinite anticoagulation. In such patients, the cumulative benefit of preventing recurrent VTE must be weighed against the cumulative risk of treatment-related hemorrhage. Reduced-dose extended treatment may improve this balance in selected patients (Agnelli et al., 2013b; Weitz et al., 2017), but it does not completely eliminate bleeding and may not be appropriate for individuals with a particularly high risk of recurrence. Consequently, a substantial unmet need remains for anticoagulant strategies capable of maintaining efficacy while producing less disruption of physiological hemostasis.

### 2.4 Rationale for new anticoagulant targets

The persistence of bleeding complications during treatment with thrombin and FXa inhibitors has stimulated the search for anticoagulant targets that may contribute predominantly to pathological thrombus formation and propagation while playing a less critical role in normal hemostasis. Factor XI (FXI) and its activated form, Factor XIa (FXIa), have emerged as particularly attractive targets within this context (Hsu et al., 2021; Greco et al., 2023).

FXI contributes to the amplification and maintenance of thrombin generation and may be especially important for the growth and stabilization of pathological thrombi. Observational evidence from individuals with congenital FXI deficiency, together with experimental studies, suggests that reduced FXI activity may confer protection against venous and arterial thrombosis, whereas the associated bleeding phenotype is generally milder and less predictable than that observed in deficiencies of coagulation factors involved in the common pathway (Hsu et al., 2021; Greco et al., 2023). However, FXI is not irrelevant to hemostasis, and bleeding can occur, particularly after trauma or surgery involving tissues with high fibrinolytic activity. Therefore, the potential safety advantage of FXI inhibition should not be interpreted as complete freedom from bleeding risk.

Several pharmacological approaches have been developed to inhibit FXI or FXIa, including monoclonal antibodies, small-molecule inhibitors, and antisense oligonucleotides (Greco et al., 2023). Early clinical studies have provided proof of concept that FXI-directed therapy can prevent thrombosis. In patients undergoing total knee arthroplasty, the monoclonal antibody abelacimab reduced postoperative VTE compared with enoxaparin at selected doses, with a low incidence of clinically relevant bleeding (Verhamme et al., 2021). These findings support the hypothesis that FXI inhibition may provide effective antithrombotic protection with a more limited effect on physiological hemostasis, although evidence from postoperative thromboprophylaxis cannot be directly extrapolated to the long-term treatment or secondary prevention of VTE.

At present, FXI and FXIa inhibitors should be considered investigational agents rather than established alternatives to DOACs. Their eventual clinical role will depend on the results of adequately powered phase III trials, direct or indirect comparisons with current anticoagulants, the identification of appropriate doses, and the evaluation of bleeding, recurrent thrombosis, drug interactions, reversibility, procedural management, and long-term safety (Hsu et al., 2021; Greco et al., 2023; Verhamme et al., 2021). It will also be necessary to determine whether their greatest value lies in broad anticoagulant use or in selected populations with simultaneously high thrombotic and hemorrhagic risks.

The development of FXI-directed therapies reinforces the importance of individualized anticoagulant treatment. Clinical phenotypes, comorbidities, biomarkers, and prediction models may ultimately help identify not only which patients require prolonged anticoagulation, but also which coagulation target provides the most favorable net clinical benefit. Factor XI inhibition should therefore not yet be viewed as a universal replacement for DOACs, but as an emerging strategy that may complement current treatments and address some of their residual limitations.

## 3 Clinical phenotypes and populations with unmet anticoagulant needs

### 3.1 Cancer-associated and incidental pulmonary embolism

Cancer-associated thrombosis (CAT) represents one of the most challenging scenarios in contemporary anticoagulant therapy because patients simultaneously exhibit an increased risk of recurrent venous thromboembolism (VTE) and major bleeding. The prothrombotic state associated with malignancy results from complex interactions between tumor cells, inflammatory pathways, endothelial dysfunction, platelet activation, anticancer therapies, and patient-related factors. Consequently, anticoagulant treatment requires continuous reassessment throughout the course of the underlying disease rather than a fixed therapeutic approach (Raskob et al., 2018; Young et al., 2018; Agnelli et al., 2020; Key et al., 2023).

The availability of direct oral anticoagulants (DOACs) has substantially expanded therapeutic options for CAT. Randomized clinical trials have demonstrated that selected DOACs are at least as effective as low-molecular-weight heparin (LMWH) in preventing recurrent VTE, although an increased incidence of gastrointestinal and genitourinary bleeding has been observed in patients with luminal gastrointestinal or unresected genitourinary malignancies (Raskob et al., 2018; Young et al., 2018; Agnelli et al., 2020; Key et al., 2023). Current international guidelines therefore recommend an individualized approach to anticoagulant selection based on tumor type, bleeding risk, renal function, potential drug-drug interactions with anticancer therapies, life expectancy, patient preferences, and treatment adherence (Lyman et al., 2021).

Incidental pulmonary embolism (IPE) has become increasingly common owing to the widespread use of contrast-enhanced computed tomography during cancer staging and follow-up. Although frequently asymptomatic, several studies have demonstrated that patients with incidental PE experience rates of recurrent VTE, major bleeding, and mortality comparable to those observed in symptomatic pulmonary embolism. Accordingly, current guidelines recommend managing most patients with incidental PE according to the same therapeutic principles adopted for symptomatic events, provided that the expected benefit of anticoagulation outweighs the individual bleeding risk (Peris et al., 2021; Lyman et al., 2021).

Large prospective registries have further improved the understanding of anticoagulant therapy in this population. In particular, data from the RIETE Registry have confirmed that cancer patients with incidental PE have a clinical course similar to those with symptomatic PE and have highlighted the complexity of balancing recurrent thrombosis against hemorrhagic complications in routine clinical practice (Peris et al., 2021). These real-world observations complement randomized trials by including patients frequently excluded from pivotal studies, such as those with advanced age, frailty, renal dysfunction, metastatic disease, or multiple competing comorbidities.

Despite the remarkable progress achieved with DOAC therapy, cancer-associated thrombosis continues to represent one of the clinical settings in which the therapeutic window remains particularly narrow. Anticoagulant selection, dose intensity, and treatment duration should therefore be continuously individualized according to the dynamic evolution of both thrombotic and hemorrhagic risks.

Whether emerging FXI/FXIa-directed therapies can improve the benefit–risk profile of anticoagulation in cancer-associated thrombosis remains to be established in adequately powered randomized clinical trials (Hsu et al., 2021; Greco et al., 2023; Verhamme et al., 2021).

### 3.2 Older and frail patients

The progressive aging of the population has profoundly changed the epidemiology of venous thromboembolism (VTE), making older adults one of the largest groups requiring anticoagulant therapy. However, advanced age should not be considered an isolated determinant of treatment decisions. Frailty, multimorbidity, cognitive impairment, functional dependence, polypharmacy, renal dysfunction, malnutrition, and recurrent falls frequently coexist and collectively influence both thrombotic and hemorrhagic risk. Consequently, chronological age alone inadequately reflects the biological complexity of older patients with VTE (Heit, 2015; Ortel et al., 2020; Stevens et al., 2021; Clegg et al., 2013).

Although direct oral anticoagulants (DOACs) have demonstrated a favorable benefit-risk profile compared with vitamin K antagonists in the general VTE population, evidence in severely frail older adults remains relatively limited because these patients were underrepresented in pivotal randomized clinical trials (van Es et al., 2014; Schulman et al., 2009; EINSTEIN Investigators et al., 2010; Investigators et al., 2012; Agnelli et al., 2013a; Investigators et al., 2013; Agnelli et al., 2013b; Weitz et al., 2017). Real-world registries have therefore become particularly valuable for understanding anticoagulant effectiveness and safety in routine clinical practice. These observational data consistently show that advanced age is associated with increased mortality, recurrent VTE, and bleeding, emphasizing the importance of individualized therapeutic decisions rather than uniform treatment strategies (Mastroiacovo et al., 2019; Fernández-Capitán et al., 2020; Peris et al., 2021; Bikdeli et al., 2021; Dubois-Silva et al., 2023; Siddiqui et al., 2024; Avnery et al., 2020; Mora et al., 2023; Golemi et al., 2020).

Frailty itself has emerged as an independent predictor of adverse outcomes beyond chronological age. Reduced physiological reserve, chronic inflammation, sarcopenia, impaired mobility, and diminished renal function contribute to a narrower therapeutic window during anticoagulation. In addition, polypharmacy substantially increases the likelihood of clinically relevant drug-drug interactions, while cognitive impairment and reduced treatment adherence may further compromise the effectiveness and safety of DOAC therapy. These factors frequently coexist, making comprehensive geriatric assessment an increasingly important component of anticoagulant management.

The decision to continue anticoagulation beyond the initial treatment phase is particularly challenging in frail patients. On one hand, discontinuation exposes patients to recurrent VTE, which carries substantial morbidity and mortality. On the other hand, indefinite anticoagulation progressively increases cumulative bleeding risk. Consequently, treatment duration should not rely exclusively on whether the index event was provoked or unprovoked, but should also incorporate dynamic clinical variables including frailty, functional status, life expectancy, comorbidity burden, renal function, previous bleeding, and patient preferences (Ortel et al., 2020; Konstantinides et al., 2019; Stevens et al., 2021).

Emerging biomarkers may further improve individualized risk assessment in older patients. Data from the RIETE Registry suggest that baseline hemoglobin concentration, blood cellular indices, D-dimer levels, and machine-learning prediction models provide complementary prognostic information regarding bleeding and recurrent VTE during anticoagulant therapy (Siddiqui et al., 2024; Avnery et al., 2020; Mora et al., 2023). More recently, lipid-related biomarkers have also been proposed as potential indicators of bleeding vulnerability during long-term anticoagulation, suggesting that biological susceptibility may contribute to hemorrhagic risk beyond drug exposure alone (Siniscalchi et al., 2025a).

The growing number of older and frail patients requiring prolonged anticoagulation highlights one of the most important unmet needs in contemporary VTE management. Future anticoagulant strategies should aim not only to preserve antithrombotic efficacy but also to further reduce bleeding complications in biologically vulnerable patients.

### 3.3 Chronic kidney disease

Chronic kidney disease (CKD) is one of the most important determinants of both thrombotic and bleeding risk in patients with venous thromboembolism (VTE). Progressive renal impairment is associated with a prothrombotic milieu characterized by chronic inflammation, endothelial dysfunction, platelet activation, oxidative stress, and impaired fibrinolysis. At the same time, uremia promotes platelet dysfunction and alterations in primary hemostasis, resulting in a paradoxical coexistence of increased thrombotic susceptibility and enhanced bleeding tendency. This dual biological vulnerability makes anticoagulant management particularly challenging in patients with impaired renal function (Ortel et al., 2020; Konstantinides et al., 2019; Stevens et al., 2021; Wattanakit and Cushman, 2009).

Renal function also has major pharmacological implications because all currently available direct oral anticoagulants (DOACs) undergo renal elimination to varying degrees. Dabigatran is the most dependent on renal clearance, whereas apixaban has the lowest proportion of renal elimination among currently available DOACs. Consequently, progressive deterioration of kidney function may lead to drug accumulation, prolonged anticoagulant activity, and an increased risk of bleeding if treatment is not appropriately adjusted (van Es et al., 2014; Schulman et al., 2009; EINSTEIN Investigators et al., 2010; Investigators et al., 2012; Agnelli et al., 2013a; Investigators et al., 2013).

Patients with advanced CKD were largely excluded from pivotal randomized trials evaluating DOACs for the treatment of VTE, resulting in limited high-quality evidence for this population. Current recommendations therefore rely on subgroup analyses, pharmacokinetic studies, observational data, and expert consensus rather than definitive randomized evidence. Periodic reassessment of renal function is essential throughout anticoagulant therapy because kidney function frequently changes during follow-up, particularly in older and frail patients with multiple comorbidities (Ortel et al., 2020; Konstantinides et al., 2019; Stevens et al., 2021; Wattanakit and Cushman, 2009).

Renal dysfunction also influences decisions regarding treatment duration. Patients with CKD experience higher rates of recurrent VTE, major bleeding, hospitalization, and mortality than individuals with preserved renal function. Consequently, the decision to extend anticoagulation beyond the initial treatment period requires individualized assessment that integrates renal function with age, frailty, hemoglobin levels, previous bleeding, concomitant medications, cancer status, and patient preferences rather than relying on a single clinical variable.

Real-world evidence has confirmed that renal impairment substantially modifies the balance between thrombotic protection and bleeding during anticoagulant therapy. These findings emphasize that individualized anticoagulant management should include serial assessment of renal function rather than a single baseline measurement, particularly in patients receiving long-term treatment (Mastroiacovo et al., 2019; Fernández-Capitán et al., 2020; Peris et al., 2021; Bikdeli et al., 2021; Dubois-Silva et al., 2023; Siddiqui et al., 2024; Avnery et al., 2020; Mora et al., 2023; Golemi et al., 2020).

Ultimately, SSPE represents a clinical scenario in which anticoagulant therapy frequently approaches the limits of its therapeutic window, emphasizing the need for individualized assessment of the expected absolute benefits and harms of treatment.

### 3.4 Subsegmental pulmonary embolism

Subsegmental pulmonary embolism (SSPE) has emerged as an increasingly frequent clinical entity following the widespread adoption of multidetector computed tomography pulmonary angiography (CTPA). Improved imaging resolution has increased the detection of isolated subsegmental thrombi, many of which are identified in patients with mild symptoms or even incidentally during diagnostic evaluation for other conditions. Consequently, the optimal management of SSPE remains one of the most debated issues in contemporary anticoagulant therapy.

Although SSPE generally involves a lower thrombotic burden than more proximal pulmonary embolism, its clinical significance should not be underestimated. The decision to initiate anticoagulant therapy requires careful evaluation of the probability of a true thromboembolic event, the presence of concomitant deep vein thrombosis, cardiopulmonary reserve, active cancer, persistent provoking factors, previous VTE, and individual bleeding risk. In selected low-risk patients without proximal deep vein thrombosis and with a high risk of bleeding, structured clinical surveillance may represent an acceptable alternative to anticoagulation. However, this approach requires careful patient selection and close follow-up (Konstantinides et al., 2019; Stevens et al., 2021).

Real-world evidence has provided important insights into the prognosis of SSPE. Data from the RIETE Registry demonstrated that patients with symptomatic SSPE experience rates of recurrent VTE, major bleeding, and mortality that are not negligible during anticoagulant treatment. Although outcomes are generally more favorable than those observed in patients with more central pulmonary embolism, the differences are smaller than previously expected, suggesting that SSPE should not automatically be regarded as a benign condition (Fernández-Capitán et al., 2020).

The management of SSPE illustrates one of the principal limitations of current anticoagulant therapy. In many patients, the expected absolute benefit of anticoagulation is relatively modest, whereas the potential consequences of major bleeding remain clinically relevant. Consequently, treatment decisions should rely on individualized assessment rather than anatomical clot location alone. Clinical characteristics, frailty, active cancer, previous VTE, renal function, concomitant deep vein thrombosis, and patient preferences should all contribute to the therapeutic decision-making process.

The uncertainty surrounding SSPE management also highlights the need for more refined risk stratification tools capable of simultaneously estimating thrombotic recurrence and bleeding risk. Biomarkers, prediction models, and machine-learning approaches may contribute to improving patient selection in the future, thereby avoiding both unnecessary anticoagulation and undertreatment (Siddiqui et al., 2024; Avnery et al., 2020; Mora et al., 2023).

Ultimately, SSPE represents a clinical scenario in which anticoagulant therapy frequently approaches the limits of its therapeutic window.

### 3.5 Patients at high bleeding risk

Patients with venous thromboembolism (VTE) who are simultaneously at high risk of bleeding represent one of the greatest challenges in anticoagulant therapy. Although anticoagulation remains the cornerstone of VTE treatment, the occurrence or anticipated risk of major bleeding frequently limits treatment initiation, influences dose intensity, shortens treatment duration, or leads to premature discontinuation. Consequently, clinical decision-making requires continuous balancing of thrombotic protection against hemorrhagic complications rather than the application of fixed therapeutic algorithms (Ortel et al., 2020; Konstantinides et al., 2019; Stevens et al., 2021).

Bleeding risk is determined by multiple interacting factors rather than a single clinical characteristic. Advanced age, frailty, chronic kidney disease, active cancer, anemia, previous bleeding, thrombocytopenia, liver disease, concomitant antiplatelet therapy, and polypharmacy frequently coexist and collectively increase hemorrhagic risk. Importantly, many of these variables are dynamic and may substantially change during long-term anticoagulant therapy, emphasizing the need for periodic reassessment rather than a single baseline evaluation.

Among rare conditions, hereditary hemorrhagic telangiectasia (HHT) represents a paradigmatic example of the complexity of anticoagulant management. Although HHT is primarily characterized by recurrent mucosal bleeding due to vascular malformations, affected patients remain susceptible to venous thromboembolism and therefore may still require anticoagulant treatment. Data from the RIETE Registry demonstrated that anticoagulation is feasible in selected patients with HHT, although bleeding complications remain more frequent than in the general VTE population, requiring individualized therapeutic decisions and close clinical follow-up (Riera-Mestre et al., 2019).

Major bleeding occurring during anticoagulant therapy frequently creates a difficult therapeutic dilemma because interruption of anticoagulation exposes patients to recurrent thromboembolism, whereas continuation may result in further hemorrhagic complications. In selected circumstances, temporary placement of an inferior vena cava (IVC) filter may be considered when anticoagulation is absolutely contraindicated or must be interrupted because of ongoing major bleeding. However, available evidence suggests that IVC filters should be regarded as temporary adjunctive measures rather than substitutes for anticoagulant therapy, and anticoagulation should be resumed as soon as clinically feasible (Mellado et al., 2019).

The management of patients at high bleeding risk also highlights the limitations of currently available bleeding prediction models. Several bleeding-risk assessment models have been proposed in patients receiving anticoagulant therapy for VTE, including VTE-BLEED and the RIETE bleeding score. These tools can support identification of patients at increased hemorrhagic risk, but their discrimination is generally moderate and performance may vary across populations, particularly in older, frail, cancer, and multimorbid patients. Bleeding-risk scores should therefore complement rather than replace individualized clinical assessment and should not be used in isolation to withhold anticoagulation when the expected thrombotic benefit remains substantial (Klok et al., 2016).

Emerging evidence suggests that biological markers may further improve bleeding risk stratification. Baseline hemoglobin concentration has consistently been associated with major bleeding during anticoagulant therapy, while recent observations indicate that lipid metabolism, particularly low-density lipoprotein cholesterol (LDL-C), may also contribute to individual bleeding susceptibility. These findings support the concept that bleeding risk depends not only on anticoagulant exposure but also on patient-specific biological vulnerability (Siniscalchi et al., 2025a).

Patients with persistently elevated bleeding risk represent an important population for evaluating anticoagulant strategies that interfere less with physiological hemostasis. Whether FXI/FXIa inhibition can provide such an advantage without compromising antithrombotic efficacy remains indication-dependent and requires confirmation in randomized trials (Hsu et al., 2021; Greco et al., 2023; Verhamme et al., 2021).

### 3.6 Clinical settings in which DOACs are not preferred

Although direct oral anticoagulants (DOACs) have become the preferred anticoagulant strategy for most patients with venous thromboembolism (VTE), important clinical scenarios remain in which alternative anticoagulant approaches are recommended or should be carefully considered. These situations illustrate the current limitations of available anticoagulants and emphasize that individualized treatment extends beyond simple drug selection.

Antiphospholipid syndrome (APS) represents the best-established example. Randomized clinical trials and observational studies have demonstrated an increased risk of recurrent thrombotic events, particularly arterial thrombosis, among high-risk APS patients treated with DOACs compared with vitamin K antagonists. Consequently, current international guidelines recommend vitamin K antagonists as the preferred long-term anticoagulant therapy in patients with triple-positive APS or previous arterial thrombotic events, whereas DOACs should generally be avoided in these settings (Ortel et al., 2020; Konstantinides et al., 2019; Stevens et al., 2021).

Pregnancy constitutes another important limitation of current DOAC therapy. Because all currently available DOACs cross the placenta and clinical safety data remain insufficient, low-molecular-weight heparin remains the anticoagulant of choice throughout pregnancy and during the postpartum period. Similarly, DOACs are generally not recommended during breastfeeding owing to the limited evidence regarding neonatal safety (Ortel et al., 2020; Konstantinides et al., 2019; Stevens et al., 2021).

Patients with severe renal impairment also require particular attention. Although selected DOACs may be used in carefully selected individuals with moderate renal dysfunction, evidence remains limited in patients with advanced chronic kidney disease or dialysis dependence because these populations were largely excluded from pivotal randomized trials. In these circumstances, anticoagulant selection should be individualized according to renal function, bleeding risk, expected treatment duration, and clinical experience (Ortel et al., 2020; Konstantinides et al., 2019; Stevens et al., 2021; van Es et al., 2014; Schulman et al., 2009; EINSTEIN Investigators et al., 2010; Investigators et al., 2012; Agnelli et al., 2013a; Investigators et al., 2013).

Another challenging scenario involves patients receiving medications that strongly inhibit or induce cytochrome P450 enzymes or P-glycoprotein transporters. Clinically significant drug-drug interactions may substantially alter DOAC plasma concentrations, increasing either bleeding or thrombotic risk. Careful review of concomitant medications therefore represents an essential component of individualized anticoagulant management, particularly in patients with cancer, transplantation, epilepsy, or chronic infectious diseases.

Mechanical prosthetic heart valves represent a well-established contraindication to DOAC therapy. Although these patients generally fall outside the spectrum of VTE management, this example illustrates that direct inhibition of thrombin or Factor Xa is not universally applicable across all thrombotic conditions. Likewise, patients with unusual thrombotic disorders or highly complex clinical presentations frequently require individualized anticoagulant strategies supported by multidisciplinary expertise.

These clinical scenarios collectively demonstrate that anticoagulant therapy cannot be regarded as a one-size-fits-all intervention. Rather, anticoagulant selection should reflect the biological characteristics of the patient, the underlying thrombotic mechanism, competing hemorrhagic risks, and the pharmacological properties of the available agents. In this context, the development of anticoagulants targeting alternative components of the coagulation cascade, such as Factor XI or activated Factor XI, may further expand therapeutic options for patients in whom current DOACs are suboptimal or contraindicated. However, whether these emerging agents will overcome the limitations of existing anticoagulants remains to be established by ongoing phase III clinical trials (Hsu et al., 2021; Greco et al., 2023; Verhamme et al., 2021).

Overall, these clinical scenarios illustrate that individualized anticoagulant management is increasingly required to balance thrombotic protection against bleeding risk. The principal patient populations in whom personalized therapeutic strategies may provide the greatest benefit are summarized in Table 1.

**TABLE 1:** Clinical scenarios requiring individualized anticoagulant therapy in patients with venous thromboembolism.

Clinical scenario	Main thrombotic concern	Main bleeding concern	Current therapeutic challenges	Potential future role of Factor XI inhibition
Cancer-associated thrombosis	High recurrence risk	Gastrointestinal and genitourinary bleeding	Drug interactions, tumor heterogeneity, prolonged treatment	Potential relevance of FXI/FXIa inhibition—investigational
Older and frail patients	Persistent VTE risk	Frailty, falls, polypharmacy, renal dysfunction	Dynamic benefit-risk assessment	Potential population for future evaluation; no established clinical role
Chronic kidney disease	Hypercoagulability	Drug accumulation, platelet dysfunction	Dose adjustment and limited evidence in severe CKD	Potential relevance requires dedicated clinical evaluation
Subsegmental pulmonary embolism	Low-to-intermediate recurrence risk	Bleeding may outweigh benefit in selected patients	Patient selection for anticoagulation	No established role; requires prospective evaluation
High bleeding risk	Recurrent VTE after treatment interruption	Previous bleeding, anemia, HHT, concomitant therapies	Difficult balance between efficacy and safety	Potential population of interest, but efficacy and safety require randomized confirmation

Legend. Major clinical scenarios in which individualized anticoagulant management is required because thrombotic and bleeding risks coexist. These populations represent potential candidates for future anticoagulant strategies aimed at improving the net clinical benefit, including Factor XI, inhibition.

### 3.7 From current personalization to precision anticoagulation

Personalized anticoagulant therapy should distinguish between factors that currently modify clinical management and emerging variables that remain investigational. At present, anticoagulant selection and duration can be individualized according to the nature of the index VTE, recurrence risk, active cancer, renal and hepatic function, bleeding history, concomitant medications, pregnancy, antiphospholipid syndrome, adherence, and patient preferences. Reduced-dose DOAC regimens during extended treatment represent another established form of treatment individualization in appropriately selected patients. In contrast, biomarkers such as LDL-C and blood cellular indices, as well as machine-learning prediction models and FXI/FXIa-directed treatment selection, remain investigational. These approaches may refine future risk stratification but should not currently determine anticoagulant choice, dose, or duration in isolation.

## 4 Biomarkers and prediction models for personalized anticoagulant therapy

Biomarkers and prediction tools relevant to personalized anticoagulation can serve different purposes, including estimation of VTE recurrence, assessment of bleeding susceptibility, characterization of the individual haemostatic phenotype, and prediction of response to anticoagulant therapy. The markers discussed below were selected according to their potential ability to influence, either currently or in the future, decisions regarding anticoagulant choice, intensity, duration, or follow-up. This section is therefore not intended as a comprehensive review of all prognostic biomarkers in acute VTE. In particular, cardiac troponins and natriuretic peptides are well-established components of acute pulmonary embolism risk stratification but primarily inform early prognostic assessment and management rather than individualized selection or duration of anticoagulant therapy (Stevens et al., 2021).

### 4.1 D-dimer

D-dimer is the most extensively investigated biomarker for estimating the risk of recurrent venous thromboembolism (VTE) after completion of anticoagulant therapy. Persistently elevated D-dimer levels following treatment discontinuation have consistently been associated with an increased risk of recurrence, whereas normal values identify patients with a more favorable prognosis (Avnery et al., 2020). However, D-dimer should not be interpreted in isolation. Current evidence supports its integration with clinical variables, including whether the index event was provoked or unprovoked, age, sex, bleeding risk, and comorbidities. Consequently, D-dimer represents one component of a broader individualized approach to treatment duration rather than a standalone decision-making tool. As novel anticoagulant strategies become available, biological markers such as D-dimer may also contribute to selecting patients who derive the greatest net clinical benefit from prolonged anticoagulation. Inherited thrombophilia represents another component of recurrence-risk assessment, although its implications for treatment duration vary substantially according to the specific abnormality and clinical context. Common heterozygous factor V Leiden and prothrombin G20210A variants generally provide limited incremental information for determining anticoagulant duration, whereas deficiencies of natural anticoagulants, including antithrombin, protein C, and protein S, may identify selected patients with a more persistent thrombotic predisposition. Thrombophilia testing should therefore be selective and interpreted together with the circumstances of the index VTE, family history, recurrence risk, and expected consequences for management rather than used as an isolated determinant of anticoagulant duration (Middeldorp et al., 2023).

### 4.2 Blood cellular indices

Routine hematological parameters have emerged as inexpensive and widely available biomarkers capable of improving prognostic assessment in patients with VTE. Indices derived from the complete blood count, including red cell distribution width, mean platelet volume, neutrophil-to-lymphocyte ratio, platelet-to-lymphocyte ratio, and systemic inflammatory indices, have all been associated with mortality, recurrent VTE, and bleeding in observational studies. Analysis of the RIETE Registry further demonstrated that blood cellular indices provide incremental prognostic information beyond conventional clinical variables, supporting their integration into future risk stratification strategies (Siddiqui et al., 2024). Although these markers are not sufficiently validated to guide anticoagulant selection independently, they may contribute to identifying patients with increased biological vulnerability.

### 4.3 Hemoglobin

Among routinely available laboratory parameters, baseline hemoglobin has consistently demonstrated one of the strongest associations with bleeding during anticoagulant therapy. Low hemoglobin should not simply be regarded as a marker of anemia but rather as an indicator of underlying clinical vulnerability reflecting frailty, chronic inflammation, renal dysfunction, occult malignancy, or previous bleeding. More recently, analysis of the RIETE Registry confirmed that baseline hemoglobin independently predicts major bleeding during anticoagulant treatment across different clinical presentations of VTE, reinforcing its potential role in individualized bleeding risk assessment (Siddiqui et al., 2024; Siniscalchi et al., 2025b). Serial evaluation of hemoglobin during follow-up may further improve recognition of patients whose bleeding risk progressively changes over time.

### 4.4 LDL-cholesterol

Growing evidence suggests that biological determinants of bleeding extend beyond traditional clinical risk factors. Recent data from the RIETE Registry demonstrated that lower baseline low-density lipoprotein cholesterol (LDL-C) concentrations were independently associated with an increased risk of major bleeding during anticoagulant therapy for VTE (Siniscalchi et al., 2025a). Although the underlying mechanisms remain incompletely understood, these findings support the hypothesis that lipid metabolism may influence vascular integrity and bleeding susceptibility. If confirmed by external validation studies, LDL-C could become an additional biomarker contributing to individualized assessment of the benefit-risk balance during long-term anticoagulation.

### 4.5 Global haemostasis assays and thrombin generation

Global haemostasis assays provide a complementary approach to conventional biomarkers by assessing the integrated functional capacity of the coagulation system rather than individual circulating components. Among these approaches, thrombin generation testing has attracted particular interest because it captures the combined influence of procoagulant and anticoagulant mechanisms. Importantly, substantial inter-individual variability in thrombin generation has been demonstrated at fixed concentrations of different anticoagulant drugs, challenging the assumption that standardized anticoagulant exposure necessarily produces a uniform biological response (Bloemen et al., 2013).

Thrombin generation has also been investigated as a predictor of recurrent VTE. In a prospective cohort of patients after a first spontaneous VTE, lower peak thrombin generation identified patients at lower risk of recurrence after anticoagulant withdrawal (Hron et al., 2006). Similarly, increased thrombin generation measured in the presence of thrombomodulin was associated with a higher risk of recurrent VTE (Tripodi et al., 2008). More recent evidence suggests that thrombin-generation parameters may also provide information on individual biological responses to DOAC exposure and bleeding susceptibility (Shaw et al., 2025).

Global haemostasis assays have also been incorporated into interventional anticoagulation research. Notably, endogenous thrombin potential was used as the primary laboratory endpoint in the RAPS trial comparing rivaroxaban with warfarin in patients with thrombotic antiphospholipid syndrome (Cohen et al., 2016). However, thrombin-generation testing is not currently established for routine adjustment of DOAC dose or treatment duration. Pre-analytical and analytical variability, lack of universally accepted reference ranges and therapeutic thresholds, and limited prospective evidence demonstrating improved clinical outcomes remain important barriers to implementation. Nevertheless, functional assessment of the net haemostatic phenotype represents a conceptually relevant direction for precision anticoagulation and warrants further prospective investigation.

### 4.6 Machine learning

Machine-learning approaches have been investigated as potential tools for improving risk prediction in VTE by integrating large numbers of clinical and laboratory variables and modelling complex non-linear relationships. However, their incremental value over conventional clinical prediction models remains uncertain. In a RIETE-based analysis, machine-learning algorithms provided only modest improvements over traditional approaches, illustrating both the possibilities and current limitations of these methods (Mora et al., 2023). More broadly, available models are heterogeneous, external validation remains limited, and methodological limitations and risk of bias frequently restrict their generalizability (Teo et al., 2026).

Accordingly, machine-learning models should currently be regarded as investigational tools rather than established instruments for personalized anticoagulant management. Their ability to improve patient outcomes or to identify differential benefit from specific anticoagulant agents, doses, or treatment durations has not been prospectively demonstrated. Future studies should prioritize external validation, prospective impact assessment, and demonstration of clinically meaningful improvement over established prediction strategies (Teo et al., 2026).

A summary of currently available biomarkers and their potential contribution to personalized anticoagulant management is provided in Table 2.

**TABLE 2:** Established and emerging tools supporting individualized risk assessment and personalized anticoagulant therapy in venous thromboembolism.

Biomarker/tool	Main information provided	Potential clinical application	Current clinical status
D-dimer	Residual thrombotic activity and risk of recurrent VTE after anticoagulant withdrawal	May contribute to assessment of recurrence risk and decisions regarding duration of anticoagulation in selected patients	Clinically established in selected recurrence-risk assessment strategies; should not be used as a stand-alone determinant of treatment duration
Inherited thrombophilia	Underlying inherited predisposition to thrombosis	May contribute to recurrence-risk assessment in selected patients, particularly in high-risk thrombophilias such as antithrombin, protein C, or protein S deficiency	Established diagnostic testing with selective, context-dependent use; common heterozygous factor V Leiden and prothrombin G20210A variants generally have limited impact on treatment duration when considered in isolation
Cardiac troponin and NT-proBNP	Myocardial injury and right ventricular strain in acute pulmonary embolism	Early prognostic stratification and identification of patients at increased risk of short-term adverse outcomes	Clinically established for acute PE risk stratification; not currently used to select anticoagulant type or duration
Bleeding-risk scores	Integrated clinical estimation of hemorrhagic risk during anticoagulation	Identification of patients requiring closer surveillance and correction of modifiable bleeding-risk factors	Clinically available; predictive discrimination is generally moderate and scores should complement, rather than replace, individualized clinical assessment
Hemoglobin	Anemia and underlying biological/clinical vulnerability associated with bleeding risk	Bleeding-risk assessment and longitudinal clinical monitoring during anticoagulant therapy	Established clinical risk marker, but not a stand-alone tool for anticoagulant selection, dose, or duration
Blood cellular indices	Systemic inflammation, disease severity, and biological vulnerability	Potential refinement of prognostic and bleeding-risk stratification	Investigational; observational evidence predominates and prospective treatment-guidance studies are lacking
LDL cholesterol	Potential biological susceptibility to bleeding	Possible future refinement of bleeding-risk assessment during anticoagulation	Investigational; association with bleeding is observational and requires external validation before clinical implementation
Thrombin generation/global haemostasis assays	Integrated functional haemostatic phenotype and biological response to anticoagulant exposure	Assessment of recurrence risk, anticoagulant response, and potentially bleeding susceptibility	Investigational; promising functional approach but limited by assay variability, lack of standardized therapeutic thresholds, and absence of prospective evidence supporting treatment adjustment
Factor XI activity	Individual FXI-dependent thrombotic susceptibility	Potential future identification of biological phenotypes relevant to FXI/FXIa-directed treatment	Investigational as a treatment-selection biomarker; elevated FXI is associated with increased VTE risk, whereas FXI deficiency is associated with lower VTE risk
Machine-learning models	Integrated multivariable prediction incorporating complex and potentially non-linear relationships	Potential future decision support for recurrence and bleeding-risk assessment	Investigational; incremental predictive improvement is generally modest, external validation is limited, and clinical utility remains unproven

Legend. Biomarkers and prediction tools relevant to personalized anticoagulation differ substantially in their level of clinical validation and potential impact on treatment decisions. D-dimer, cardiac biomarkers, conventional clinical variables, and bleeding-risk scores already contribute to risk assessment in selected clinical settings, although none should independently determine anticoagulant choice or duration. In contrast, blood cellular indices, LDL, cholesterol, thrombin-generation assays, Factor XI, activity as a treatment-selection biomarker, and machine-learning models remain investigational. Importantly, prognostic association does not necessarily imply treatment-guidance utility, and prospective validation and clinical impact studies are required before emerging biomarkers or prediction tools can be incorporated into routine precision-anticoagulation strategies.

## 5 Factor XI/Factor XIa inhibition: an emerging strategy in anticoagulant therapy

### 5.1 Biological rationale

Despite the remarkable advances achieved with direct oral anticoagulants (DOACs), bleeding remains the principal limitation of long-term anticoagulant therapy. Current anticoagulants reduce thrombotic events by inhibiting thrombin or activated Factor X (FXa), both of which play fundamental roles in physiological hemostasis. Consequently, suppression of thrombosis inevitably increases the risk of bleeding, particularly in older patients, individuals with multiple comorbidities, active cancer, renal dysfunction, or other conditions associated with increased biological vulnerability (van Es et al., 2014; Schulman et al., 2009; EINSTEIN Investigators et al., 2010; Investigators et al., 2012; Agnelli et al., 2013a; Investigators et al., 2013; Agnelli et al., 2013b; Weitz et al., 2017; Hsu et al., 2021; Greco et al., 2023; Verhamme et al., 2021).

Factor XI (FXI) has emerged as an attractive therapeutic target because it occupies a distinct position within the coagulation cascade. In contrast to thrombin and FXa, FXI contributes predominantly to thrombus amplification and stabilization rather than to the initiation of physiological hemostasis. Experimental models and observations in individuals with congenital FXI deficiency have demonstrated reduced thrombotic risk associated with a generally mild and variable bleeding phenotype, suggesting that selective inhibition of FXI may partially dissociate antithrombotic efficacy from impairment of normal hemostasis (Hsu et al., 2021; Greco et al., 2023).

Human epidemiological evidence further supports the relationship between FXI activity and thrombotic susceptibility. In the Leiden Thrombophilia Study, plasma FXI levels above the 90th percentile were associated with approximately a twofold higher risk of deep vein thrombosis, with evidence of a dose-response relationship (Meijers et al., 2000). Conversely, individuals with FXI deficiency have a substantially lower incidence of VTE than those with normal FXI activity (Preis et al., 2017). Together, these observations provide an important epidemiological foundation for pharmacological FXI/FXIa inhibition. However, whether measurement of FXI activity itself can serve as a biomarker to select patients for FXI-directed therapy remains unknown and should currently be considered investigational.

These observations have generated the hypothesis that anticoagulants targeting FXI or activated FXI (FXIa) may preserve protection against venous thromboembolism while reducing clinically relevant bleeding. Although this concept remains under clinical investigation, it represents one of the most important paradigm shifts in anticoagulant pharmacology over the last decade.

### 5.2 Pharmacological strategies

Several pharmacological approaches have been developed to inhibit the FXI pathway, reflecting the growing interest in this therapeutic target. These include monoclonal antibodies directed against FXI or FXIa, antisense oligonucleotides that reduce hepatic FXI synthesis, and orally active small-molecule FXIa inhibitors. Despite their different mechanisms of action, these agents are being developed with the aim of attenuating FXI-dependent thrombin amplification while potentially exerting less interference with physiological hemostasis than conventional anticoagulants. Whether this pharmacological rationale translates into a consistently improved clinical benefit–risk profile remains indication-specific and has not yet been established (Hsu et al., 2021; Greco et al., 2023).

Among monoclonal antibodies, abelacimab is a long-acting, fully human antibody that binds FXI and prevents its activation. In the phase IIb AZALEA–TIMI 71 trial in patients with atrial fibrillation, abelacimab markedly reduced major or clinically relevant non-major bleeding compared with rivaroxaban; however, this trial was primarily designed to assess bleeding and was not powered to establish non-inferior thromboembolic efficacy (Ruff et al., 2025). Phase III evaluation of abelacimab in cancer-associated thrombosis has included the ASTER trial, comparing abelacimab with apixaban (NCT05171049), and the MAGNOLIA trial, comparing abelacimab with dalteparin in patients with gastrointestinal or genitourinary cancers (NCT05171075). Both studies are currently listed on ClinicalTrials.gov as terminated following a sponsor decision. In the absence of published phase III efficacy and safety results from these trials, no conclusions regarding the benefit–risk profile of abelacimab in cancer-associated VTE should be drawn from their termination status alone. Asundexian was associated with substantially fewer major bleeding events than apixaban in OCEANIC-AF but failed to provide adequate protection against stroke or systemic embolism, whereas OCEANIC-STROKE subsequently demonstrated a reduction in recurrent ischemic stroke without a significant increase in major bleeding when asundexian was added to antiplatelet therapy after non-cardioembolic ischemic stroke or high-risk transient ischemic attack (Sharma et al., 2026). Milvexian remains under phase III evaluation in atrial fibrillation and secondary stroke prevention within the LIBREXIA programme, whereas LIBREXIA-ACS was discontinued following a prespecified interim analysis indicating that the trial was unlikely to meet its primary efficacy endpoint. Collectively, these findings indicate that the clinical efficacy of FXI/FXIa inhibition is highly dependent on the underlying thrombotic setting, dose, and comparator, and caution against considering FXI inhibition as a uniformly safer substitute for conventional anticoagulation.

### 5.3 Current clinical evidence

Early proof-of-concept evidence for FXI/FXIa inhibition was generated predominantly in thromboprophylaxis after major orthopedic surgery, where several phase II studies demonstrated effective prevention of postoperative VTE with generally low bleeding rates (Verhamme et al., 2021; Weitz et al., 2021; Piccini et al., 2022). More recently, phase III programmes in atrial fibrillation and secondary stroke prevention have provided indication-specific evidence, with markedly divergent efficacy findings. In this setting, several phase II studies demonstrated that FXI inhibition effectively reduces postoperative VTE while maintaining a favorable bleeding profile compared with conventional anticoagulant therapy (Verhamme et al., 2021; Weitz et al., 2021; Piccini et al., 2022).

Beyond orthopedic thromboprophylaxis, the clinical development of FXI/FXIa inhibitors has produced heterogeneous and, in some settings, contrasting results. In atrial fibrillation, the phase III OCEANIC-AF trial was terminated prematurely because asundexian was associated with a substantially higher incidence of stroke or systemic embolism than apixaban, despite producing significantly fewer major bleeding events. This finding provides an important proof of principle that greater preservation of hemostasis does not necessarily ensure sufficient antithrombotic efficacy.

Conversely, more favorable results have emerged in secondary prevention after non-cardioembolic ischemic stroke. In the phase III OCEANIC-STROKE trial, asundexian 50 mg once daily, added to antiplatelet therapy, reduced recurrent ischemic stroke compared with placebo (6.2% vs. 8.4%; HR 0.74, 95% CI 0.65–0.84), while the incidence of major bleeding was similar between groups (1.9% vs. 1.7%; HR 1.10, 95% CI 0.85–1.44) (Sharma et al., 2026).

The contrasting results of OCEANIC-AF and OCEANIC-STROKE also raise an important mechanistic question regarding the clinical settings in which FXIa inhibition provides sufficient antithrombotic intensity. In atrial fibrillation, prevention of cardioembolic events may require more profound suppression of coagulation than that achieved with the tested asundexian regimen, whereas non-cardioembolic ischemic stroke involves a more heterogeneous interplay between platelet activation and coagulation. In the latter setting, selective attenuation of FXIa-dependent thrombin amplification may therefore provide additional antithrombotic protection without substantially increasing bleeding. These interpretations remain hypothesis-generating, but the divergent phase III results argue against assuming a uniform class effect and support indication-specific evaluation of FXI/FXIa inhibition (Piccini et al., 2025; Capodanno et al., 2025).

The development of milvexian provides another example of this uncertainty. Phase III evaluation continues in atrial fibrillation and secondary stroke prevention within the LIBREXIA programme, whereas the LIBREXIA-ACS trial was discontinued after an interim analysis indicated that the study was unlikely to meet its primary efficacy endpoint. Similarly, the phase III ASTER and MAGNOLIA trials evaluating abelacimab in cancer-associated VTE are currently registered as terminated following a sponsor decision. As phase III efficacy and safety results from these studies have not yet been published, their termination should not be interpreted as evidence of either benefit or harm. Taken together, current evidence does not support considering FXI/FXIa inhibition as a class effect that uniformly combines preserved efficacy with reduced bleeding. Rather, the available results suggest a potentially wider therapeutic window in selected clinical settings, accompanied by insufficient efficacy in others. No FXI/FXIa inhibitor is currently established as an alternative to DOACs for the treatment or secondary prevention of VTE, and its eventual role will require indication-specific evidence from adequately powered randomized trials. The major FXI/FXIa inhibitors currently in clinical development and their key clinical findings are summarized in Table 3.

**TABLE 3:** Major Factor XI/Factor XIa inhibitors in clinical development and key clinical findings.

Agent	Mechanism	Main indication studied	Development phase/status	Main clinical findings
Abelacimab	Monoclonal antibody against FXI/FXIa	Orthopedic VTE prevention; AF; cancer-associated VTE	Phase II–III	Superior to enoxaparin at selected doses after knee arthroplasty; substantially less bleeding than rivaroxaban in AF; CAT phase III ASTER/MAGNOLIA terminated
Asundexian	Oral small-molecule FXIa inhibitor	AF; secondary stroke prevention	Phase III	OCEANIC-AF: less bleeding but higher stroke/systemic embolism vs apixaban; OCEANIC-STROKE: reduced recurrent ischemic stroke without significant increase in major bleeding
Milvexian	Oral small-molecule FXIa inhibitor	AF; ischemic stroke/TIA; ACS	Phase III	Phase III AF/stroke programmes ongoing; LIBREXIA-ACS discontinued for futility
FXI antisense oligonucleotides	Reduction of hepatic FXI synthesis	Orthopedic thromboprophylaxis	Phase II	Proof-of-concept efficacy; slower onset and prolonged pharmacodynamic effect

Legend. Summary of the principal Factor XI/Factor XIa (FXI/FXIa)-directed agents currently evaluated in clinical development. Mechanisms of action, major clinical indications, development phase or status, and key findings from relevant clinical trials are reported. Available evidence remains heterogeneous across indications, and no FXI/FXIa, inhibitor is currently established as a replacement for direct oral anticoagulants in the treatment or secondary prevention of venous thromboembolism. Trial status and clinical evidence were updated through August 2026.

### 5.4 Future perspectives

The development of FXI inhibitors should not be viewed as an attempt to replace DOACs universally. Instead, these agents may ultimately expand therapeutic options for selected patients in whom the balance between recurrent thrombosis and bleeding remains particularly unfavorable despite optimal contemporary management.

These populations represent clinically plausible candidates for future evaluation of FXI/FXIa-directed strategies; however, differential benefit in these subgroups has not been established and should not currently guide treatment selection. In this context, biomarkers, real-world evidence, and machine-learning prediction models may become increasingly important for identifying patients most likely to benefit from individualized anticoagulant strategies.

Further randomized phase III trials directly comparing FXI inhibitors with current DOACs are needed before these agents can be incorporated into routine clinical practice. Future studies should evaluate not only recurrent VTE and major bleeding but also patient-reported outcomes, treatment adherence, cost-effectiveness, and long-term safety. Rather than representing a uniform replacement for current anticoagulants, FXI/FXIa inhibition may ultimately occupy indication-specific therapeutic niches in which reduced interference with hemostasis can be achieved without compromising antithrombotic efficacy. Identifying these settings remains a central objective of ongoing clinical development.

## 6 Beyond anticoagulation: the potential role of statins

Statins have anti-inflammatory, endothelial-protective and antithrombotic properties that provide a biological rationale for a possible adjunctive role in VTE. Observational studies, including analyses from the RIETE Registry, have reported associations between statin use and lower mortality in patients with VTE, although effects on recurrent VTE have been inconsistent (Siniscalchi et al., 2023; Siniscalchi et al., 2024; Siniscalchi et al., 2022; Undas et al., 2005). However, these findings are observational and susceptible to residual confounding, and randomized evidence supporting statins as a VTE-specific therapeutic strategy is lacking. Statins should therefore be prescribed according to established cardiovascular indications rather than specifically for the treatment or secondary prevention of VTE. Their potential influence on thrombotic and bleeding outcomes remains hypothesis-generating and warrants further prospective investigation. Nevertheless, their pleiotropic effects and favorable safety profile make them attractive candidates for future studies exploring integrated strategies aimed at reducing both thrombotic complications and overall mortality (Siniscalchi et al., 2019).

## 7 Conclusion

Venous thromboembolism has evolved from a disease managed with standardized anticoagulant protocols to a condition requiring increasingly individualized therapeutic decisions. Although direct oral anticoagulants have transformed VTE management by simplifying treatment while maintaining efficacy and improving safety compared with vitamin K antagonists, important unmet clinical needs remain, particularly in patients with advanced age, frailty, chronic kidney disease, active cancer, and increased bleeding risk.

Clinical phenotypes, routinely available biomarkers, and real-world evidence increasingly support a precision medicine approach to anticoagulant therapy. Rather than relying on a single clinical variable, future management will likely integrate biological vulnerability, patient characteristics, and dynamic risk assessment to optimize anticoagulant selection, treatment intensity, and duration.

Factor XI/FXIa inhibition represents an important emerging approach in anticoagulant pharmacology. Phase III evidence has demonstrated clinical benefit in selected indications, such as secondary prevention after non-cardioembolic ischemic stroke, while inadequate efficacy has been observed in others, including atrial fibrillation with asundexian. These findings argue against a uniform class effect and support indication- and agent-specific evaluation. Importantly, no FXI/FXIa inhibitor is currently established as an alternative to DOAC therapy for the treatment or secondary prevention of VTE.
